# Incorporation of Bone Marrow Cells in Pancreatic Pseudoislets Improves Posttransplant Vascularization and Endocrine Function

**DOI:** 10.1371/journal.pone.0069975

**Published:** 2013-07-18

**Authors:** Christine Wittig, Matthias W. Laschke, Claudia Scheuer, Michael D. Menger

**Affiliations:** Institute for Clinical & Experimental Surgery, University of Saarland, Homburg/Saar, Germany; University of Bremen, Germany

## Abstract

Failure of revascularization is known to be the major reason for the poor outcome of pancreatic islet transplantation. In this study, we analyzed whether pseudoislets composed of islet cells and bone marrow cells can improve vascularization and function of islet transplants. Pancreatic islets isolated from Syrian golden hamsters were dispersed into single cells for the generation of pseudoislets containing 4×10^3^ cells. To create bone marrow cell-enriched pseudoislets 2×10^3^ islet cells were co-cultured with 2×10^3^ bone marrow cells. Pseudoislets and bone marrow cell-enriched pseudoislets were transplanted syngeneically into skinfold chambers to study graft vascularization by intravital fluorescence microscopy. Native islet transplants served as controls. Bone marrow cell-enriched pseudoislets showed a significantly improved vascularization compared to native islets and pseudoislets. Moreover, bone marrow cell-enriched pseudoislets but not pseudoislets normalized blood glucose levels after transplantation of 1000 islet equivalents under the kidney capsule of streptozotocin-induced diabetic animals, although the bone marrow cell-enriched pseudoislets contained only 50% of islet cells compared to pseudoislets and native islets. Fluorescence microscopy of bone marrow cell-enriched pseudoislets composed of bone marrow cells from GFP-expressing mice showed a distinct fraction of cells expressing both GFP and insulin, indicating a differentiation of bone marrow-derived cells to an insulin-producing cell-type. Thus, enrichment of pseudoislets by bone marrow cells enhances vascularization after transplantation and increases the amount of insulin-producing tissue. Accordingly, bone marrow cell-enriched pseudoislets may represent a novel approach to increase the success rate of islet transplantation.

## Introduction

Clinical islet transplantation has been proposed as an ideal therapeutic strategy for the treatment of type 1 diabetes mellitus, especially for patients suffering from glycemic lability and hypoglycemia despite adequate insulin treatment [1]. In fact, since the adoption of the Edmonton Protocol in 1999, several studies reported the successful transplantation of pancreatic islets into patients, resulting in a constant insulin independency for at least one year [2]. The advantages of islet transplantation compared to whole organ pancreas grafting are the reduced operative trauma and the reduced number of complications, as only endocrine tissue is replaced. However, islet transplantation bears also several disadvantages. The most restricting factor is the large number of islets necessary to achieve normoglycemia after transplantation [3]. For successful transplantation most patients require islets prepared from two or more donor pancreases [1], [2]. The functional capacity of transplanted islets is estimated to be only 20–40% of that in non-diabetic persons [4].

The major reason for the failure of islet transplantations is thought to be the insufficient revascularization of the grafts. During the first 3 to 6 days after transplantation islet grafts lack an initial vascular supply and solely depend on oxygen and nutrient transport by diffusion [5]. During this critical period, hypoxia may induce both apoptosis and necrosis in the central β-cell-containing compartment of the islets, resulting in a decreased graft survival and function [6]. The function of surviving islet grafts may further be hampered by a reduced microvascular perfusion. This view is supported by experiments, demonstrating a signifi-cantly lower vascular density of islet grafts compared to endogenous pancreatic islets [7].

Based on this knowledge, new strategies have to be developed, aiming at an acceleration of islet graft vascularization and an increase of endocrine function. The incorporation of bone marrow cells (BMC) into pseudoislets (PI) may be capable of increasing both the posttransplant angiogenic response and the functional β-cell mass. Recently, Penko et al. [8] showed *in vitro* that it is possible to compose mosaic pseudoislets which contain pancreatic interspersed vasculogenic endothelial progenitor cells (EPC). The effect of BMC or EPC interspersed in pseudoislets on posttransplant vascularization and *in vivo* endocrine function, however, is completely unknown. Therefore, we created pseudoislets containing BMC and pancreatic islet cells (BMC-PI) and studied for the first time whether those BMC-enriched pseudoislets are capable of improving the process of vascularization and increasing the endocrine function after free transplantation.

## Research Design and Methods

### Animals

Six- to eight-week-old inbred Syrian golden hamsters were used for the microcirculatory studies, and 9–15-week-old animals for diabetes induction. The animals were housed one per cage and had free access to tap water and standard pellet food (Altromin, Lage, Germany). The experiments were conducted in accordance with the German legislation on protection of animals and the NIH Guidelines for the Care and Use of Laboratory Animals (NIH Publication #85-23 Rev. 1985). They were specifically approved by the governmental animal care committee of the Saarland, Germany, (permit number: 29/06).

### Isolation of pancreatic islets and bone marrow cells

**Figure 1:**
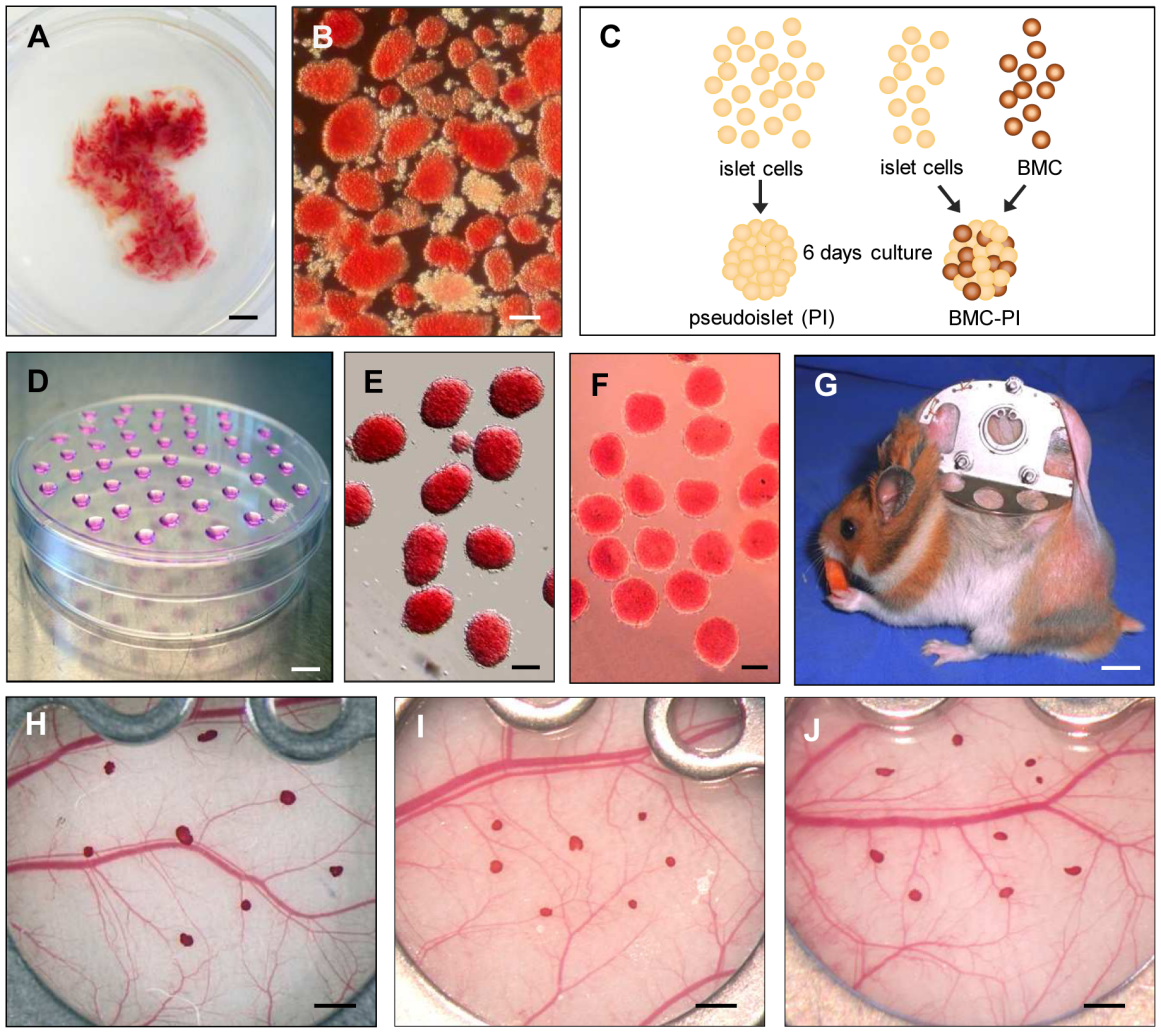
Isolation, *in vitro*-generation and transplantation of NI, PI and BMC-PI. **A**: Freshly excised pancreas of a Syrian golden hamster after collagenase injection, stained with neutral red. Scale bar: 5 mm. **B**: Freshly isolated pancreatic islets. Scale bar: 100 µm. **C**: Schematic representation of the *in vitro* generation of PI and BMC-PI. **D**: Hanging drop culture for the generation of PI or BMC-PI, drop volume 20 µL. Scale bar: 10 mm. **E**: Freshly harvested PI at day 5 day after generation. Scale bar: 100 µm. **F**: Freshly harvested BMC-PI at day 5 after generation. Scale bar: 100 µm. **G**: Syrian golden hamster equipped with a dorsal skinfold chamber. Scale bar: 10 mm. **H**–**J**: Neutral red stained native pancreatic islets (**H**), PI (**I**) and BMC-PI (**J**) directly after transplantation onto the host striated muscle tissue within the dorsal skinfold chamber. Scale bar: 1 mm.

Animals were anesthetized by intraperitoneal injection of pentobarbital sodium (50 mg/kg body weight (bw)). After laparotomy, the pancreatic duct was injected with collagenase (0.8 mg/mL, type V, SERVA, Heidelberg, Germany) and pancreatic native islets (NI) were isolated as described previously in detail [9]. The islets were handpicked and transferred to a Petri dish with fresh Dulbecco's modified Eagle's medium (DMEM; PAA Laboratories GmbH, Cölbe, Germany) containing 10% fetal calf serum, 100 U/mL penicillin and 0.1 mg/mL streptomycin (Fig. 1).

For the isolation of BMC, the femurs and tibias were bilaterally harvested from the islet donor animals. The bone marrow antrum was punctured with a syringe and rinsed thoroughly with 5 mL of isolation buffer (PBS (PAA), ACD-A 1% (Fresenius, Bad Homburg, Germany), 20 mM HEPES-buffer (SERVA)). The resulting cell suspension was collected, cleaned and stored in the culture medium until further processing.

### Generation of PI and BMC-PI

For generation of PI, freshly isolated islets were dispersed into single cells by enzymatic digestion with trypsin/EDTA (0.5%/0.2%; PAA). The cell count was adjusted to a number of 2*10^5^/mL. The cell suspension was then distributed as 20 µL drops on a Petri dish surface. The Petri dishes were inverted for subsequent culture according to the hanging-drop method [10] (Fig. 1). Culture was performed for 5–6 days in a humidified incubator at 29°C to allow reaggregation of the single cells for the generation of PI (Fig. 1). Drops for the generation of PI contained finally ∼4*10^3^ islet cells.

For generation of BMC-PI freshly isolated islets were also dispersed into single cells by enzymatic digestion with trypsin/EDTA (0.5%/0.2%; PAA). The cell count was adjusted to a number of 2*10^5^/mL. Additionally, 2*10^5^/mL of freshly isolated bone marrow cells were added to the islet cells. The cell suspension was distributed as 20 µL drops on a Petri dish, which was inverted for subsequent culture according to the hanging-drop method [10] (Fig. 1). Culture was performed for 5–6 days in a humidified incubator at 29°C to allow re-aggregation of the single cells as already done for the generation of PI (Fig. 1). Drops for the generation of BMC-PI contained ∼2*10^3^ islet cells and ∼2*10^3^ BMC.

The BMC did not attach to the surface of the Petri dishes, because the Petri dishes were inverted for culture (hanging-drop). The gravitational force prevented that the cells stayed in contact to the Petri dish surface. The lower temperature of 29°C instead of 37°C was chosen to prevent early core cell damage inside the grafts [6].

### Generation of GFP-BMC-PI

To evaluate an *in vitro* differentiation of BMC contained in the BMC-PI to an insulin-producing cell-type, additional GFP-BMC-PI were generated from murine tissue. For these experiments BMC were isolated from femurs and tibias of mice, expressing the green fluorescent protein (GFP) (C57BL/6-TgN(ACTbEGFP)1Osb, Jackson Laboratories, Jackson, Missouri). Pancreatic islets were isolated from wild-type C57BL/6 mice (Charles River, Sulzfeld Germany), dispersed in single cells and mixed to an equal amount with the GFP-BMC for hanging-drop culture, as described above. After 5–6 days the GFP-BMC-PI were handpicked and fixed in formalin for immunohistochemical analysis.

### Static glucose stimulation and insulin secretion analysis

Insulin secretion of native islets (NI) (d0 and d5) as well as PI and BMC-PI was analyzed *in vitro* by a static glucose stimulated insulin secretion assay (GSIS). For this, all islet types were first preincubated for 2 h in 37°C Krebs Ringer Buffer (KRB) containing 2.8 mM glucose. Then, the islets were handpicked as groups of 10IEQ [11] in Eppendorf tubes (n = 3 per group) and incubated in a shaking water bath for 1 h at 37°C in 500 µL KRB containing 2.8 mM glucose. After one hour 300 µL of the supernatant were collected and frozen at −20°C. The remaining supernatant was completely removed and another 500 µL of KRB containing 16.7 mM glucose were added. The specimens were then incubated for another 60 min in a shaking water bath at a temperature of 37°C. After this one-hour period, again, 300 µL of the supernatant were collected and frozen at −20°C.

Experiments were performed in triplicate. Insulin concentrations were quantified by an enzyme linked immunosorbent assay (ELISA) (rat/mouse insulin ELISA kit, Merck Millipore, Darmstadt, Germany). The stimulation index (SI) was calculated by dividing the average insulin secretion at 16.7 mM glucose by the average insulin secretion at 2.8 mM glucose.

### Preparation of the dorsal skinfold chamber

For the *in vivo* analysis of islet vascularization, we used the dorsal skinfold chamber model. The chamber technique and its implantation procedure have been described previously in detail [12]. Briefly, under intraperitoneal pentobarbital sodium anesthesia (50 mg/kg bw), two symmetrical titanium frames were implanted on the extended dorsal skinfold of the animals, so that they sandwiched the double layer of skin. One layer of skin was then removed in a circular area of 15 mm in diameter. The remaining layers, consisting of striated muscle, subcutaneous tissue and skin, were covered with a removable cover slip incorporated into one of the titanium frames (Fig. 1). After the preparation, the animals were allowed to recover from anesthesia and surgery for at least 48 h.

### Transplantation of NI, PI and BMC-PI into the dorsal skinfold chamber

For transplantation, the cover glass of the skinfold chamber was removed and 7 to 8 neutral-red-stained NI, PI or BMC-PI (diameter: 150–200 µm) were placed onto the striated muscle within the chamber (Fig. 1). After transplantation the chamber was closed by a new cover glass.

### Intravital fluorescence microscopy

For *in vivo* microscopic observations, anesthetised hamsters (pentobarbital sodium) were injected retrobulbary with 0.1 mL of 5% fluorescein isothiocyanate (FITC)-labeled dextran 150,000 (Fluka, Buchs, Switzerland). FITC-dextran was used for contrast enhancement to visualize the microvessels. Intravital microscopy was performed using a Zeiss Axiotech microscope equipped with a 100 W mercury lamp and a blue and a green filter block for epi-illumination (Zeiss, Oberkochen, Germany). The microscopic images were recorded with a charge-coupled device video camera (FK 6990-IQ; Pieper, Berlin, Germany) for off-line evaluation.

### Microcirculatory analysis

Quantitative off-line analysis of the microscopic images was performed by the computer-assisted image analysis system CapImage (Zeintl, Heidelberg, Germany). Analyses included the determination of the diameter (µm) and the area (mm^2^) of the islet grafts at day 0. During the further post-transplant time course the revascularized area of the grafts (mm^2^), the functional capillary density, i.e. the length of newly formed red blood cell (RBC)-perfused microvessels per observation area (cm/cm^2^), as well as the diameters (µm) and the RBC velocity (V_RBC_, µm/s) of these microvessels were determined [13]. Microvascular diameters and RBC velocity (V_RBC_) were determined in 20 microvessels within each individual graft. Volumetric blood flow (VQ, pL/s) of individual microvessels was calculated from V_RBC_ and diameter (d) for each microvessel as VQ  =  π*(d/2)^2^*V_RBC_/K, where K ( = 1.3) represents the Baker-Wayland factor [14], considering the parabolic velocity profile of blood cells in microvessels.

### Transplantation of NI, PI and BMC-PI under the kidney capsule

To examine the *in vivo* function, islets were transplanted under the right kidney capsule of diabetic hamsters (n = 4 animals each group). For diabetes induction, hamsters received a single intraperitoneal injection of streptozotocin (60 mg/kg bw, Fluka, Sigma-Aldrich, Taufkirchen, Germany). Seven days after streptozotocin injection, blood glucose levels were measured. Only animals with a blood glucose level higher than >11.1 mM [15] were used for the transplantation experiments. Islets were then transplanted under the right kidney capsule using a Hamilton syringe (22-G; Hamilton, Bonaduz, Switzerland). Blood glucose levels (AccuChek Compact Plus; Roche Diagnostics, Mannheim, Germany) and body weight of the animals were controlled at regular intervals over a 3-week period. At day 21 the right kidney and the pancreas were removed for histological analyses.

### Experimental protocol

#### Islet transplantation in skinfold chambers

For the *in vivo* microcirculatory analyses, NI, PI and BMC-PI were transplanted into skinfold chambers (n = 8 animals each group). Intravital fluorescence microscopy was performed directly after transplantation (d 0) as well as at days 3, 6, 10 and 14. At the end of the experiments the chamber tissue was carefully excised and processed for immunohistochemical analyses.

#### Islet transplantation under the kidney capsule

To study islet function *in vivo*, 1000 IEQ of NI, PI and BMC-PI were transplanted under the right kidney capsule (n = 4 animals each group). A fourth group of animals (NI+BMC) received 500 IEQ of native islets and an equal amount of BMC (1*10^6^ cells; n = 4). Sham-operated animals without islet transplantation served as diabetic controls (n = 4). Blood glucose and body weight of the animals were determined at day 0, 1, 3, 5, 7, 10, 14, 18 and 21 after transplantation.

### Fixation of specimens

Isolated islets as well as PI, BMC-PI and GFP-BMC-PI were fixed in HepatoQuick reagent (Roche, Mannheim, Germany), which is normally used for the determination of the prothrombin time. In detail, in a lid of an Eppendorf tube 100 µL of the HepatoQuick reagent were mixed with 50 µL human citrate plasma and 10 µL of a 10% CaCl_2_ solution to start the gelation of the mixture. After 20 min at room temperature about 15–20 islets, PI, BMC-PI or GFP-BMC-PI were placed on the gelated thromboplastin reagent. Immediately thereafter, a second layer of the mixture of the HepatoQuick reagent, the human citrate plasma and CaCl_2_ was placed over the islets. After 30 min the solidified islet-containing gel layers were removed from the Eppendorf tube lids and transferred to 4% paraformaldehyde.

### Immunohistochemistry

After 5–6 days culture NI, PI and BMC-PI were fixed in formalin and embedded in paraffin. 5 µm sections were incubated overnight at room temperature with a guinea pig polyclonal anti-insulin antibody (1∶100; Abcam, Cambridge, UK), a mouse monoclonal anti-glucagon antibody (1∶50, Abcam) and a rabbit polyclonal anti-somatostatin antibody (1∶500, Abcam). This was followed by 45 min incubation with the appropriate Cy3-conjugated secondary antibody (Dianova, Hamburg, Germany). Nuclei were counterstained with bisbenzimide (Hoechst 33258; Sigma-Aldrich). The number of insulin-, glucagon- and somatostatin-producing cells was counted and given in percent of all cells visible.

To analyze the number of remnant endothelial cells inside the different islet types, CD31 staining was performed on 5 µm sections of 5–6 days in vitro cultured NI, PI and BMC-PI with a rat polyclonal anti-mouse anti-CD31 antibody (1∶30; Dianova; overnight at room temperature). Visualization was achieved by 45 min incubation with a Cy3-labeled goat anti-rat IgG secondary antibody (1∶50; Dianova). Nuclei were stained with bisbenzimide. Specimens of GFP-BMC-PI were also analyzed for CD31-positive cells and were additionally double stained with a rat polyclonal anti-mouse anti-CD31 antibody (1∶30; Dianova) and a goat polyclonal anti-GFP antibody (1∶200; Rockland Immunochemicals, Gilbertsville, USA) overnight at room temperature. Signals were visualized with a Cy3-labeled goat anti-rat IgG secondary antibody (1∶50; Dianova) and a donkey polyclonal biotin-labeled anti-goat IgG (1∶30; Jackson Immunoresearch Europe Ltd., Suffolk, UK). Additional enhancement of the GFP signal was achieved by streptavidin-FITC (1∶50; Vector Laboratories, Burlingame, USA).

For immunohistochemical double staining of insulin and GFP in GFP-BMC-PI, 5 µm paraffin sections (n = 20) were first incubated with a rabbit polyclonal anti-insulin antibody (1∶50; Santa Cruz, Heidelberg, Germany) for 2 h at room temperature and then over night at 4°C. This was followed by incubation with a goat polyclonal anti-GFP antibody (1∶200; Rockland Immunochemicals) overnight at 4°C. A goat polyclonal Cy3-labeled anti-rabbit IgG and a rabbit polyclonal biotin-labeled anti-goat IgG (1∶30; Jackson) were used as secondary antibodies. Additional enhancement of GFP was achieved by streptavidin-FITC (1∶50; Vector Laboratories). Nuclei were counterstained with bisbenzimide. The number of insulin-, GFP- and double-positive cells was counted in 20 different GFP-BMC-PI specimens and is given in percent of all visible cells.

Cleaved caspase-3 served as an indicator of apoptotic cell death. Five µm sections of paraffin-embedded specimens of NI, PI and BMC-PI (n = 10) were incubated overnight at room temperature with a rabbit polyclonal anti-cleaved caspase-3 antibody (1∶100, New England Biolabs, Heidelberg, Germany). This antibody detects endogenous levels of the large fragment (17/19 kDa) of activated caspase-3, but not full length caspase-3. For streptavidine-biotin complex peroxidase staining, a biotinylated ready to use goat anti-rabbit IgG antibody was used (Abcam) in combination with strepatavidine 3.3′ diaminobenzidine (DAB; Sigma) as chromogen. The sections were counterstained with hemalaun. Positively stained cells were counted and given in percent of the total cell number.

From the *in vivo* experiments, skinfold chamber tissue, pancreases and graft-containing kidneys were fixed in formalin (4%) and embedded in paraffin. To study hormone production, 3 µm sections were incubated over night at room temperature with a guinea-pig polyclonal anti-insulin antibody (1∶100; Abcam). This was followed by 45 min incubation with the appropriate peroxidase-conjugated secondary antibody. DAB was used as the chromogen and hemalaun for counterstaining.

To evaluate the number of newly formed blood vessels in the grafts under the kidney capsule, specimens were additionally stained against CD31 with a rat polyclonal anti-mouse antibody (1∶100; Dianova) and were visualized with a Cy3-labeled goat anti-rat IgG secondary antibody (1∶1000; Dianova) using fluorescence microscopy. Nuclei were stained with bisbenzimide.

### Western blot analysis

Whole protein extracts of NI, PI and BMC-PI were loaded on a 10% SDS polyacrylamide gel (PAGE) and electro-blotted onto a polyvinyldifluoride membrane (0.2 µm, BioRad, Munich, Germany). The membrane was incubated for 2 h at room temperature with a rabbit polyclonal anti-insulin antibody (1∶300; Santa Cruz). Protein expression was visualized by luminol-enhanced chemiluminescence (ECL; GE-Healthcare, Freiburg, Germany) and exposure of the membranes to a blue light sensitive autoradiography film (Hyperfilm ECL, GE-Healthcare). Signals were densitometrically assessed (Geldoc, Quantity one software; Bio-Rad). To confirm equal protein loading, the same blots were reincubated with antibodies specific for the housekeeping protein GAPDH (1∶100; Santa Cruz).

### Statistical analysis

All data are expressed as means ± SEM. Data were first analyzed for normal distribution and equal variance. Because of non-normal distribution of data, non-parametric analyses were performed. Differences between the groups were evaluated by the Kruskal Wallis one way ANOVA on ranks. This was followed by the Tukey post-hoc test, including the correction of the alpha-error according to Bonferroni probabilities to compensate for multiple comparisons. P-values <0.05 were considered to indicate a significant difference.

## Results

### *In vitro* hormone expression and release of NI, PI and BMC-PI

**Figure 2:**
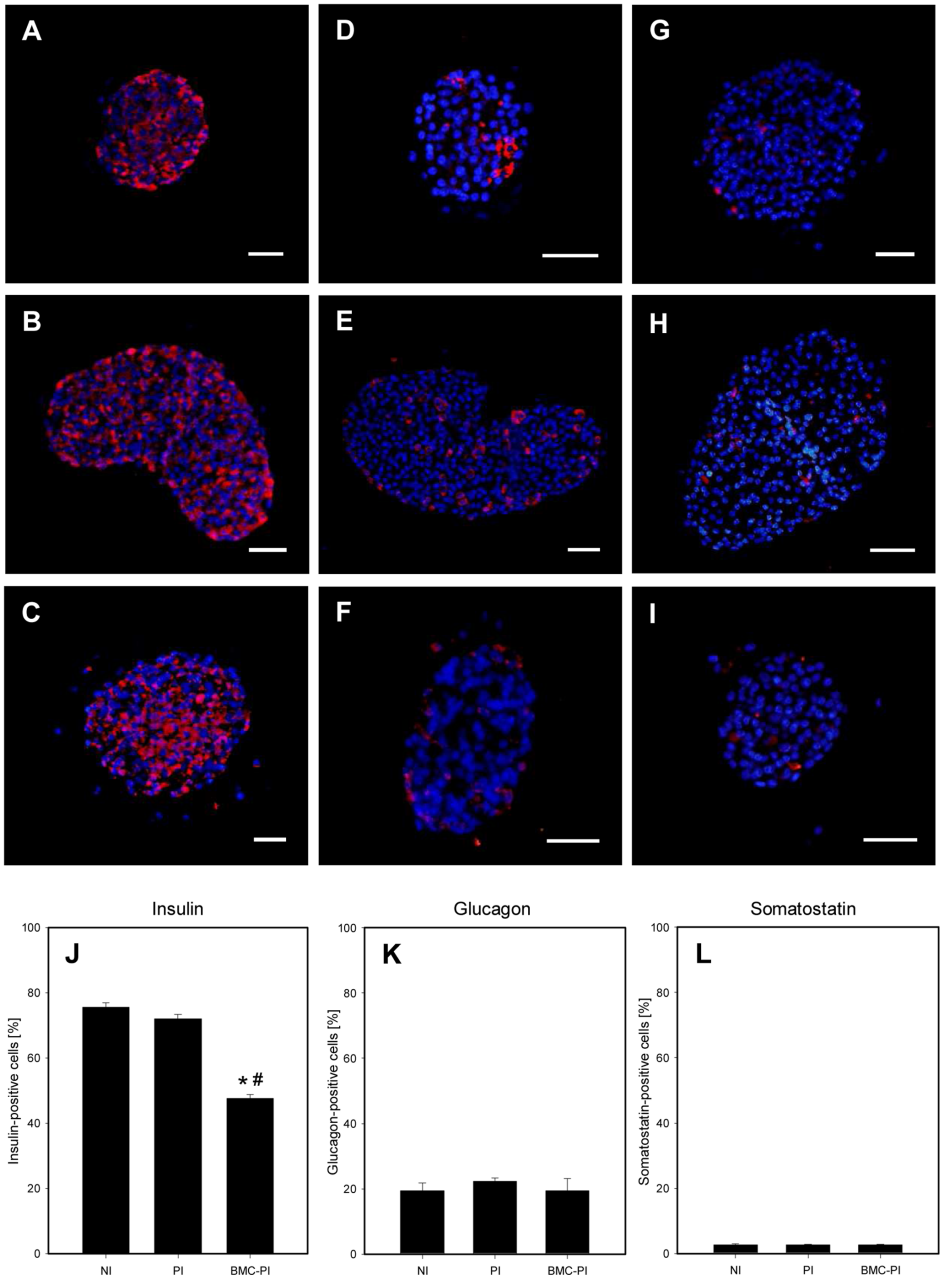
Immunohistochemical analysis of hormone production inside of NI, PI and BMC-PI. Immunohistochemical sections of *in vitro* cultured NI (**A**, **D**, **G**), PI (**B**, **E**, **H**) and BMC-PI (**C**, **F**, **I**). The sections were stained with an anti-insulin (**A–C**), an anti-glucagon (**D**–**F**) and an anti-somatostatin antibody (**G**–**I**). Scale bars: 50 µm. **J**–**L**: Number of insulin- (**J**), glucagon- (**K**) and somatostatin-positive cells (**L**) given in percent of all cells. Data are given as mean ± SEM (n = 4, data evaluated in triplicate, *p<0.05 vs. NI, ^#^p<0.05 vs. PI).

Immunohistochemical analysis of *in vitro* samples revealed that NI, PI and BMC-PI contained densely distributed insulin-positive cells, located in the center as well as in the periphery (Figs. 2A–C). In all three types of islets the majority of glucagon-positive cells were found in the periphery (Figs. 2D–F). Moreover, all of the three types of islets showed only few somatostatin-positive cells (Figs. 2G–I). As expected, NI as well as PI contained a significantly higher number of insulin-positive β-cells of 75% and 71% compared to the BMC-PI with only 48% (Fig. 2J). Further immunohistochemical staining showed ∼20% glucagon-positive cells and ∼3% somatostatin-positive cells without significant differences between NI, PI and BMC-PI (Figs. 2K and L).

**Figure 3:**
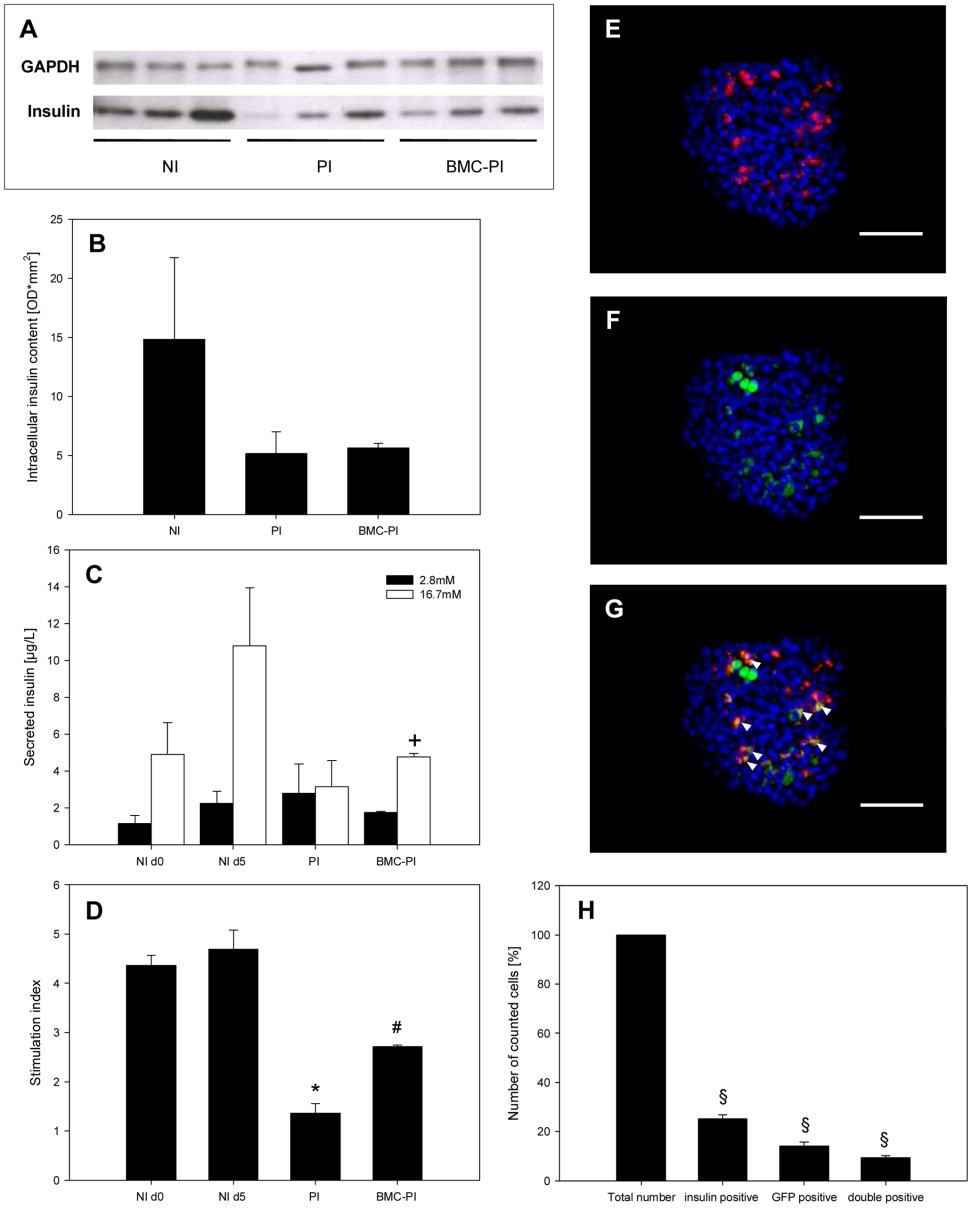
*In vitro* intracellular insulin content and secretion of NI, PI and BMC-PI. **A**, **B**: Western blot analysis of insulin protein (12 kDa) and GAPDH protein (37 kDa) expression (optical density (OD) * mm^2^) of NI, PI and BMC-PI, which were cultured for 5 days in DMEM with 10% FCS. **C**: Secreted insulin of NI d0, NI d5, PI and BMC-PI after stimulation with 2.8 mM (black) or 16.7 mM glucose (white). Data are given as mean ± SEM, (^+^p<0.05 vs. 2.8 mM glucose). **D**: Glucose-dependent stimulation index (SI) of insulin secretion of NI directly after isolation (d0) and after 5 d culture (d5) as well as PI and BMC-PI. Data are given as mean ± SEM (*p<0.05 vs. NI (d0 and d5), ^#^p<0.05 vs. BMC-PI). **E**–**G**: Immunofluorescent histological section of a 6-day *in vitro* cultured GFP-BMC-PI. The section was stained with anti-insulin (**E**) and anti-GFP (**F**). Nuclei were counterstained with bisbenzimide. **G** represents a merge of **E** and **F**. Note that some of the GFP-positive BMC show insulin expression (arrow heads). Scale bars: 50 µm. **H**: Number of insulin-, GFP- and double-positive cells inside the GFP-BMC-PI, given in percent. Data are given as mean ± SEM (^§^p<0.05 vs. total number of cells).

Western blot analysis of intracellular insulin after 5 days of culture confirmed that NI, but also PI and BMC-PI produce insulin. Of interest, NI showed higher insulin contents than PI and BMC-PI. Statistical analysis, however, did not prove a significance of this difference (Figs. 3A and B).

Analysis of insulin secretion revealed in non-cultured, freshly isolated native islets (d0) a low basal insulin secretion of 1.1 µg/L in the presence of 2.8 mM glucose and an increase to 4.9 µg/L in the presence of 16.7 mM glucose. This indicates a stimulation index of 4.4 (Figs. 3C and D). Five days cultured native islets showed a basal insulin secretion of 2.3 µg/L, which increased to 10.8 µg/L in the presence of 16.7 mM glucose. This reflects a stimulation index of 4.7. Pseudoislets (PI) showed a basal insulin secretion of 2.8 µg/L and an only slight increase to 3.2 µg/L in the presence of 16.7 mM glucose. This reflects a stimulation index of 1.4. BMC-PI showed a basal insulin secretion of 1.8 µg/L and an increase to 4.8 µg/L in the presence of 16.7 mM glucose. This indicates a stimulation index of 2.7 (Figs. 3C and D).

The immunohistochemical analysis of GFP-BMC-PI after 6 days of culture (Figs. 3E–H) showed a distinct fraction of GFP-positive BMC, which were positively stained for insulin (Figs. 3E, F and G). Quantitative analysis revealed that ∼25% of the cells were insulin-positive, ∼14% of the cells were GFP-positive, and ∼9% of the cells were double positive for GFP and insulin (Fig. 3H).

### *In vitro* analysis of endothelial cells in cultured NI, PI and (GFP)-BMC-PI

To evaluate the fraction of remnant endothelial cells inside the cultured islet clusters, CD31 staining was performed on sections of NI, PI and BMC-PI. On specimens of GFP-BMC-PI a double staining against GFP and CD31 was performed. All stainings in 5–6 days cultured NI, PI and BMC-PI as well as the double stainings in GFP-BMC-PI were found negative for CD31.

### *In vitro* analysis of apoptotic cell death in cultured NI, PI and BMC-PI

Immunohistochemical analysis of apoptotic cell death by caspase-3 staining revealed 8.2±1.7% caspase-3-positive cells in 5–6 days cultured NI, which did not differ from 7.8±1.6% and 8.2±0.6% caspase-3-positive cells in 5–6 days cultured PI and BMC-PI.

### *In vivo* analysis of post-transplant vascularization of NI, PI and BMC-PI

Analysis of islet diameters and islet areas directly after transplantation into the dorsal skinfold chambers revealed values of 310–340 µm and 0.08–0.1 mm^2^ without significant differences between NI, PI and BMC-PI. By this, it could be excluded that differences in revascularization between the groups are due to differences in transplant size.

**Figure 4:**
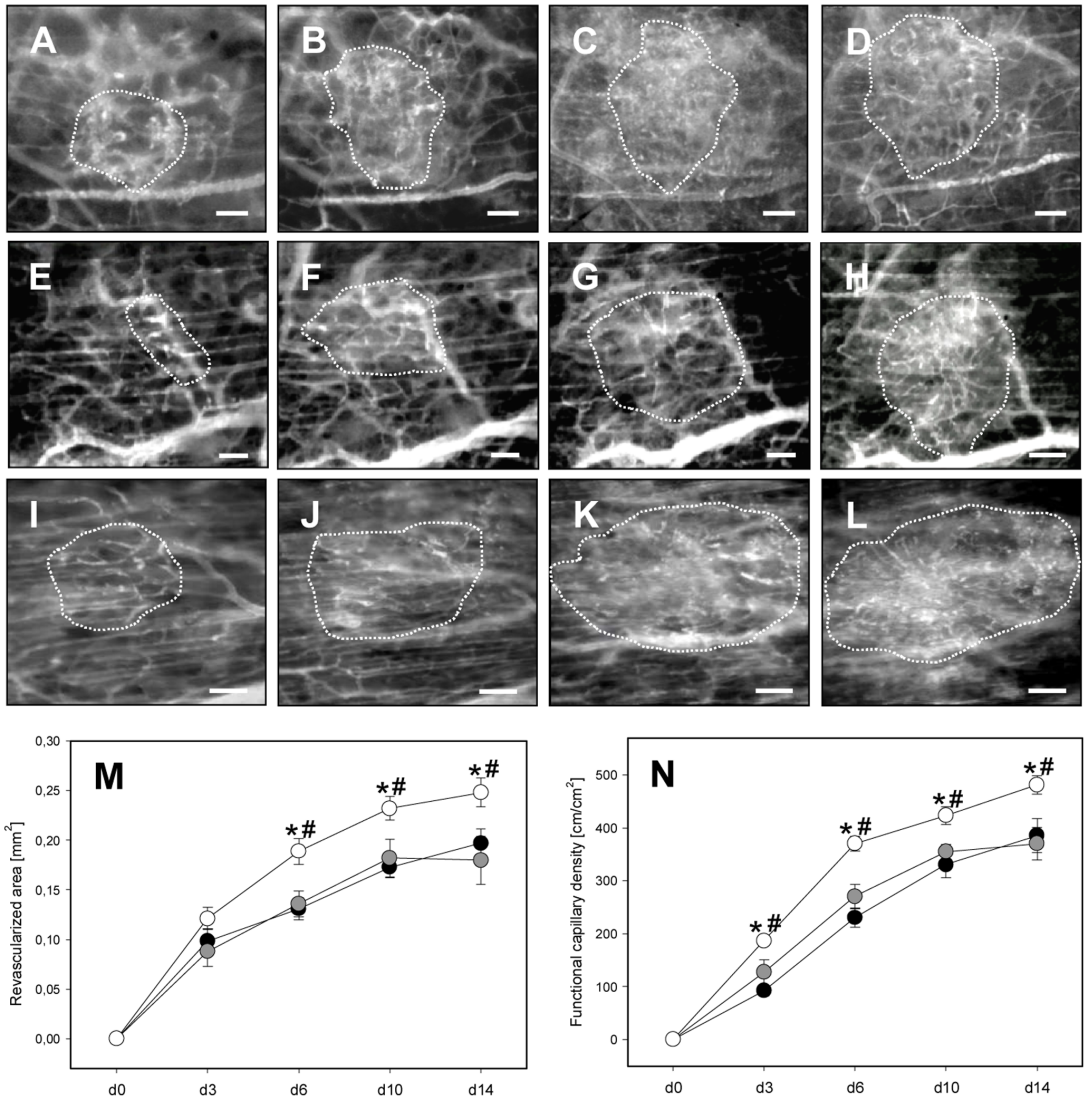
Revascularization of NI, PI and BMC-PI. **A**–**L**: Intravital microscopic images of the process of vascularization of an NI (**A**–**D**), PI (**E**–**H**) and BMC-PI (**I**–**L**) (borders marked by dotted line) at day 3 (**A, E, I**), 6 (**B, F, J**), 10 (**C, G, K**) and 14 (**D, H, L**) after transplantation into the dorsal skinfold chamber. After 14 days, the graft displays a glomerulum-like network of microvessels, which can easily be distinguished from the parallelly arranged capillaries of the host striated muscle tissue. Visualization by blue light epi-illumination with contrast enhancement by 5% FITC-labeled dextran 150,000 i.v. Scale bars: 50 µm. **M, N**: Revascularized area (**M**) and functional capillary density (**N**) of NI (black circles), PI (grey circles) and BMC-PI (white circles) directly after transplantation (d0) as well as at days 3, 6, 10 and 14. Data are given as mean ± SEM; (n = 8 per group, *p<0.05 vs. NI, ^#^p<0.05 vs. PI).

All three types of islets showed revascularization after transplantation. This included angiogenic sprouting from the host microvasculature and network formation of the newly formed blood vessels (Figs. 4A–L). Analysis of the revascularized area and the density of newly formed capillaries revealed a process of vascularization in PI similar to that observed in NI (Figs. 4M and N). Of interest, BMC-PI exhibited an improved vascularization, as indicated by a significantly increased revascularized area and a higher functional capillary density throughout the 14-day observation period (Figs. 4M and N).

Analysis of microhemodynamic parameters indicated larger microvessel diameters in BMC-PI compared to NI and PI (Table 1). Accordingly, calculated values of volumetric blood flow in microvessels of BMC-PI were also higher compared to that in microvessels of NI and PI (Table 1).

**Table 1:** Microhemodynamics in freely transplanted NI, PI and BMC-PI.

	day 3	day 6	day 10	day 14
***Microvessel diameter (µm)***				
NI	6.3±0.2	7.0±0.2	7.0±0.3	6.6±0.3
NI	7.1±0.2*	7.2±0.2	7.2±0.1	7.2±0.1
BMC-PI	7.5±0.2*	7.9±0.1* #	8.0±0.1*	7.9±0.2*
***RBC velocity (µm/s)***				
NI	92.4±5.9	134.0±15.7	155.0±20.7	132.0±17.8
PI	140.0±21.5	155.0±22.1	140.0±16.2	147.0±17.5
BMC-PI	136.0±15.9*	159.0±15.2	138.0±20.0	133.0±17.2
***Volumetric blood flow (pL/s)***				
NI	2.2±0.2	4.1±0.7	4.8±0.9	3.6±0.8
PI	4.3±0.7*	4.9±0.8	4.4±0.6	4.6±0.6
BMC-PI	4.6±0.5*	6.0±0.6* #	5.4±0.8	5.1±0.7

Diameter (µm), RBC-velocity (µm/s) and volumetric blood flow (pL/s) of newly formed microvessels within native islets (NI), pseudoislets (PI) and bone marrow cell-enriched pseudoislets (BMC-PI) at day 3, 6, 10 and 14 after transplantation into the dorsal skinfold chamber. All data are mean ± SEM.

*p<0.05 vs. NI.

# p<0.05 vs. PI.

Immunohistochemical analysis of tissue specimens at day 14 after transplantation into the dorsal skinfold chamber showed adequate intracellular insulin staining of NI, PI and BMC-PI (data not shown).

### *In vivo* analysis of post-transplant function of NI, PI and BMC-PI

**Figure 5:**
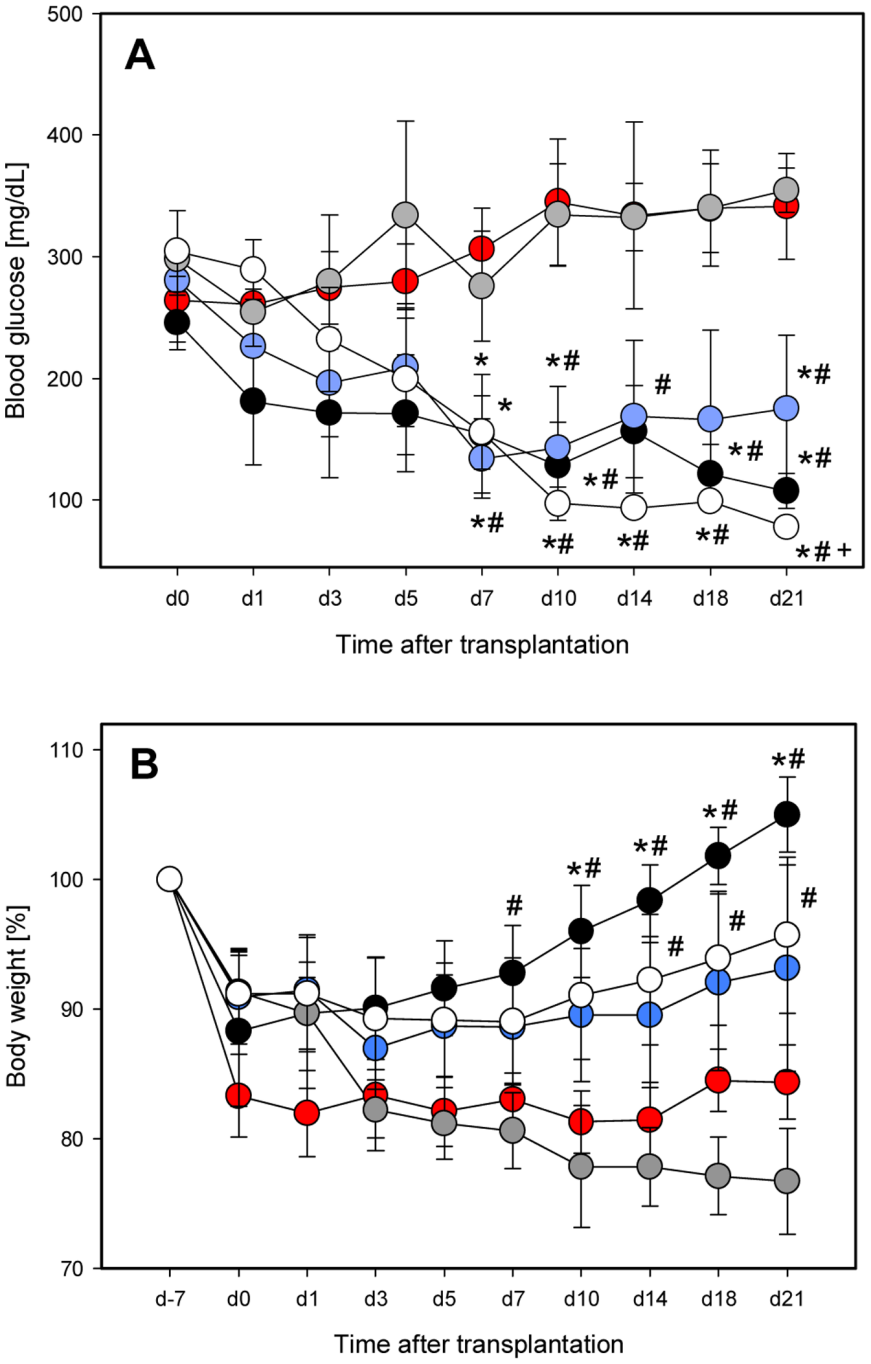
Functional outcome of islet transplantation in an *in vivo* diabetes model. Blood glucose levels (**A**) and body weight (**B**) of the animals (in percent) over the course of diabetes induction (d-7 to d0) and the 21 days after islet transplantation. Animals received NI (black circles), NI co-transplanted with BMC (blue circles), PI (grey circles) or BMC-PI (white circles) under the kidney capsule. Non-transplanted controls (red circles) served as controls. Data are given as mean ± SEM, (n = 4 per group, *p<0.05 vs. non-transplanted controls, ^#^p<0.05 vs. PI, ^+^p<0.05 vs. NI+BMC).

After transplantation of NI under the kidney capsule blood glucose levels decreased, achieving normoglycemia at day 21 with 108±14 mg/dL (Fig. 5A). In contrast, transplantation of PI under the kidney capsule was not capable of reducing blood glucose levels during the entire 21-day observation period. At day 21, blood glucose concentration was almost the same as that observed in non-transplanted controls (354±18 mg/dL vs. 342±44 mg/dL; Fig. 5A). Of interest, transplantation of BMC-PI under the kidney capsule resulted in normoglycemia (<100 mg/dL) already at day 10. Normoglycemia was found maintained until the end of the 21 day observation. The final blood glucose concentration was 78±2 mg/dL (Fig. 5A). Transplantation of half of the amount of NI together with BMC (NI+BMC) resulted also in a decline in blood glucose values over the post-transplant time course. However, normoglycemia could not be achieved during the three week experimental period as indicated by blood glucose values of 175±60 mg/dL (Fig. 5A).

Analysis of body weight showed a recovery in animals which received NI, NI+BMC and BMC-PI (Fig. 5B). In contrast, the body weight of the PI-transplanted animals did not recover over the 21-day observation period and was ∼20% below the weight at induction of diabetes. This corresponded to the changes of body weight observed in non-transplanted diabetic controls (Fig. 5B).

**Figure 6:**
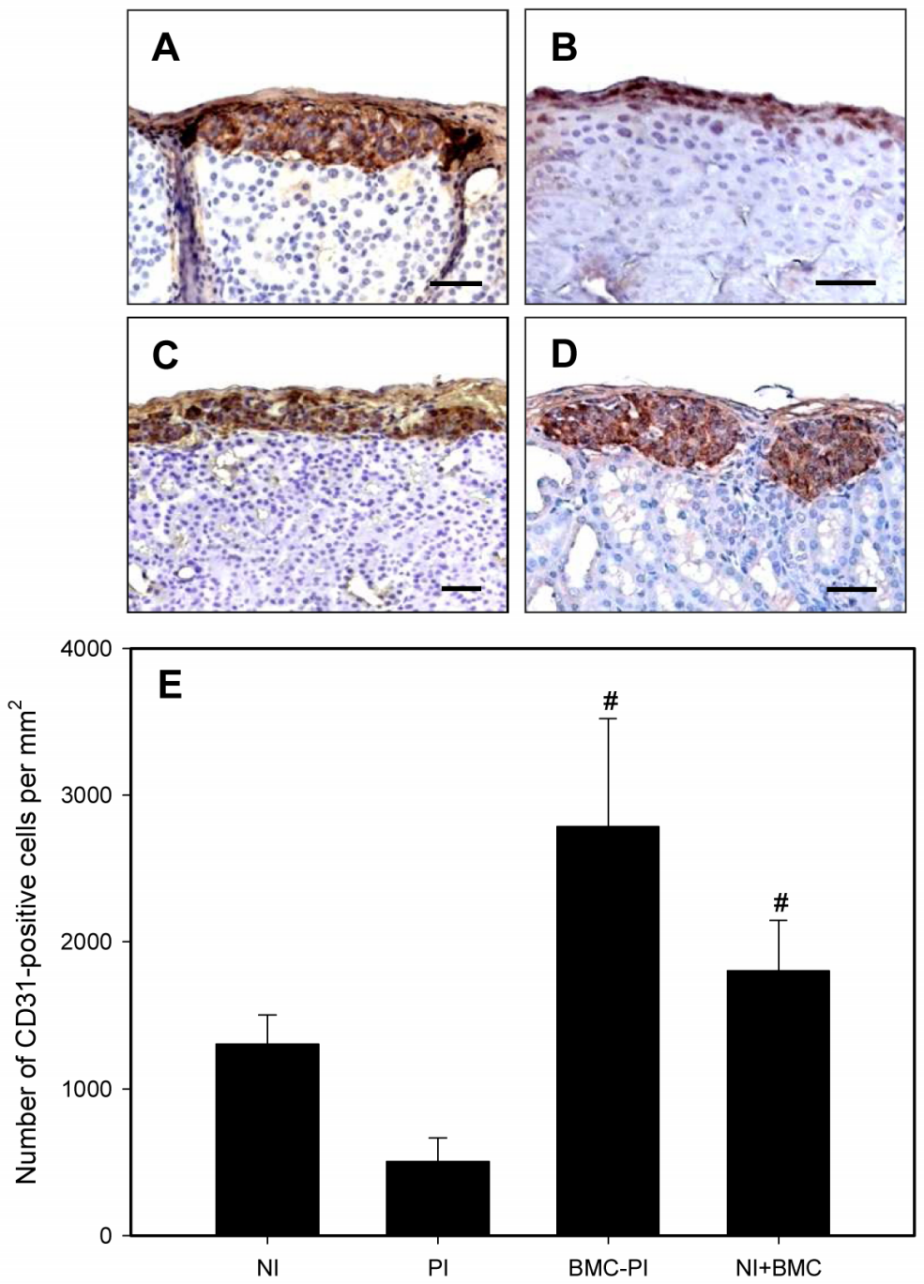
Insulin production and vessel density of NI, PI, BMC-PI and NI+BMC after transplantation. Immunohistochemical analysis of insulin expression of NI (**A**), PI (**B**), BMC-PI (**C**) and NI co-transplanted with BMC (**D**) 21 days after transplantation under the kidney capsule. Scale bars: 50 µm. **E**: Number of CD31-positive cells per mm^2^ as analyzed by immunohistochemistry in NI, PI, BMC-PI, and NI co-transplanted with BMC. Data are given as mean ± SEM, (n = 10, ^#^p<0.05 vs. PI).

Histological analysis of the pancreas of the diabetic animals showed islets with advanced vacuolization and destroyed cellular composition. Analysis of the graft bearing kidneys revealed large amounts of islet cells under the capsule. Grafts containing NI, NI+BMC and BMC-PI showed numerous insulin-positive cells (Figs. 6A, C and D). In contrast, grafts containing PI showed a reduced mass of insulin-expressing cells (Fig. 6B).

Anti-CD31 staining revealed a high density of newly formed blood vessels in transplants consisting of NI, NI+BMC and, in particular, BMC-PI. In contrast, vascularization was sparse and significantly reduced in PI grafts (Fig. 6E).

## Discussion

The major finding of the present study is that an enrichment of pseudoislets with BMC accelerates revascularization after free transplantation and improves post-transplant islet function. BMC-PI but not PI normalized blood glucose levels after transplantation into diabetic animals, although BMC-PI contained only half of the number of islet cells compared to PI or NI. Our data further indicate that the improved BMC-PI graft function is due to the accelerated revascularization. Interestingly, we also observed transdifferentiation of individual BMC to insulin-producing cells.

After enzymatic isolation pancreatic islets are completely avascular [16]. After free transplantation the process of revascularization requires 10 to 14 days to be completed [17]. During this initial period after transplantation, the islet grafts have to be supplied by oxygen diffusion. Due to their size of up to ∼300 µm, β-cells in the core of the grafts become hypoxic and undergo apoptotic and necrotic cell death [6]. This hypoxia-associated loss of islet mass during the early post-transplant period is thought to be responsible for the poor results experienced in clinical islet transplantation [18]. It may also be the cause that islets of at least two donor pancreases are needed to achieve normoglycemia after transplantation [2].

The vascularization of the freely transplanted islets is driven by the ingrowth of newly formed blood vessels from the host microvasculature [19]. In addition, it has been suggested that up to 40% of the developing microvessels within the islet grafts is formed by remnant intra-islet endothelial cells [16], [20]. However, the number of intra-islet endothelial cells has been shown to abate during culture [21], [22]. This is most probably the cause that we could not detect CD31-positive cells in specimens of 5–6 days cultured NI, PI and (GFP)-BMC-PI. Accordingly, in the present study remnant endothelial cells did not play a role in the process of revascularization of NI and PI.

Besides, we made the interesting observation that the cultured NI, PI and BMC-PI exhibited a comparable fraction of glucagon- and somatostatin-expressing cells, although BMC-PI consisted only to 50% of islet cells. This may be explained by the finding that β-cells may undergo dedifferentiation and reprogramming under certain conditions, enabling them to express also the non-β-cell hormones glucagon or somatostatin [23], [24]. In the present study these conditions may be provided by co-culture of islet cells with BMC.

Transplanted BMC-PI developed a dense glomerulum-like network of nutritive capillaries, which consisted of a similar architecture as known for NI and PI [25], [26]. However, BMC-PI showed an accelerated vascularization after transplantation and a significantly higher functional capillary density compared to NI and PI. This may be due to the fact that BMC contain significant numbers of EPC, which are known to contribute to vasculogenic processes during neovascularization [27]. Those vasculogenic BMC are also capable of enhancing angiogenic functions in islet grafts [28]. EPC represent a cell population with a CD34^+^, CD133^+^ and VEGFR-2^+^ phenotype [29]. In our study in mice, we confirmed that the bone marrow of the mice contained CD34^+^ cells, CD133^+^ cells, VEGFR-2^+^ cells and Sca-1^+^ cells (data not shown). The double expression of Sca-1 and VEGFR-2, indicative for EPC in mice, is ∼2% of the BMC fraction [30]. These EPC may have contributed to the improved revascularization of the transplanted BMC-PI.

In addition, BMC are well known to produce growth factors and cytokines to exert paracrine effects, stimulating neovascularization and regeneration [31]. Thus, BMC-released growth factors may have further contributed to the increased angiogenic response in the transplanted BMC-PI, similarly as reported from *in vitro* experiments in human islets coated with bone marrow-derived mesenchymal stem cells (MSC) and dermal microvascular endothelial cells [32].

Beside an increased functional capillary density, BMC-PI showed a significantly increased diameter of the newly formed blood vessels, and, thus, an elevated volumetric blood flow. This vasodilation may be mediated by nitric oxide, since BMC treatment has been shown to significantly improve nitric oxide bioactivity [33]. The increased blood perfusion observed in the BMC-PI transplants may have additionally contributed to the improved endocrine function.

In the present study, BMC-PI showed an adequate insulin response upon glucose stimulation *in vitro*. Further, BMC-PI showed the most rapid normalization of blood glucose in diabetic animals *in vivo*, although only 50% of islet cells were transplanted compared to NI and PI. Several factors may have contributed to this improved post-transplant function. First, acceleration of revascularization and increased blood perfusion by BMC may have prevented hypoxia-induced apoptotic or necrotic death of endocrine cells. Second, the vasculogenic BMC may not only have enhanced angiogenic functions in the islet grafts, but may also have exerted anti-inflammatory effects. Islet preparation and transplantation is associated with an increase in c-Jun NH2-terminal kinases (JNK) and pro-inflammatory Th1 cytokines, inducing early apoptotic death of islet cells after transplantation [34]–[36]. Of interest, vasculogenic BMC can activate anti-inflammatory islets survival pathways, which significantly improve islet engraftment and endocrine function [28]. Third, BMC may have transdifferentiated into insulin-producing cells. *In vitro*, we could show that individual BMC from GFP-positive mice can transdifferentiate to insulin-producing cells after co-culture and aggregation to PI with islet cells from wild-type animals. This result is supported by a report from Ianus et al. [37], demonstrating that bone marrow-derived cells can become glucose-responsive, insulin-secreting cells in islets *in vivo*. Whether transdifferentiated BMC substantially contribute to the islet graft fraction after transplantation, however, remains to be determined.

Besides transdifferentiation, BMC may also be capable of stimulating new β-cell formation. This view is supported by others, showing in streptozotocin-induced diabetic animals that intra-pancreatic homing of BMC is associated with an increase of islet mass and a normalization of blood glucose levels [38], [39]. The mechanisms of this improved islet function after BMC homing, however, remains to be determined [40].

Previous studies have analyzed whether co-transplantation of MSC with NI is capable of improving islet revascularization and engraftment [41], [42]. Rackham and coworkers [41] have shown that co-transplantation of NI and mesenchymal stem cells under the kidney capsule can reduce hyperglycemia, but does not result in normoglycemic blood glucose levels. Lu et al. [42] also co-transplanted MSC with NI and could observe by immunohistochemistry some increased insulin expression of the transplants. In the present study, the co-transplantation of BMC beneath NI only slightly increased islet vascularization. It could further reduce blood glucose levels in diabetic animals, however, also without achieving normoglycemia. In contrast, the transplantation of BMC-PI showed a significantly increased islet vascularization and a rapid normalization of blood glucose levels in diabetic animals. This indicates that the improvement of islet vascularization, engraftment and function by BMC cannot fully be achieved by just co-transplantation but requires the incorporation of the BMC into the intra-islet β-cell environment.

Taken together, our study demonstrates that the enrichment of PI with BMC improves vascularization, engraftment and function after free transplantation. Because the BMC-PI contained only half of the amount of islet cells, this strategy can be of great interest to overcome the limitations due to donor organ shortage.
